# Hypothesis: protein and RNA attributes are continuously optimized over time

**DOI:** 10.1186/s12864-019-6371-0

**Published:** 2019-12-23

**Authors:** Sidney B. Cambridge

**Affiliations:** Department of Functional Neuroanatomy, Heidelberg University, Heidelberg, Germany

**Keywords:** Protein / mRNA stability, Protein structure, Gene conservation

## Abstract

**Background:**

Little is known why proteins and RNAs exhibit half-lives varying over several magnitudes. Despite many efforts, a conclusive link between half-lives and gene function could not be established suggesting that other determinants may influence these molecular attributes.

**Results:**

Here, I find that with increasing gene age there is a gradual and significant increase of protein and RNA half-lives, protein structure, and other molecular attributes that tend to affect protein abundance. These observations are accommodated in a hypothesis which posits that new genes at ‘birth’ are not optimized and thus their products exhibit low half-lives and less structure but continuous mutagenesis eventually improves these attributes. Thus, the protein and RNA products of the oldest genes obtained their high degrees of stability and structure only after billions of years while the products of younger genes had less time to be optimized and are therefore less stable and structured. Because more stable proteins with lower turnover require less transcription to maintain the same level of abundance, reduced transcription-associated mutagenesis (TAM) would fixate the changes by increasing gene conservation.

**Conclusions:**

Consequently, the currently observed diversity of molecular attributes is a snapshot of gene products being at different stages along their temporal path of optimization.

## Background

Typical protein attributes such as abundance, stability, number of protein-protein interactions, or folded vs. unfolded regions are increasingly characterized on a global level. Large-scale analyses offer the opportunity for an unbiased characterization of such innate molecular attributes. For example, protein half-life and turnover is an attribute which has been thoroughly investigated [1–9]. However, analyses of protein turnover in a variety of organisms including bacteria [3], yeast [2, 7], mammalian cells [1, 4, 9], whole animals [5, 8], and even humans [6] have not yielded any significant insight as to why turnover values among proteins of the same cell can vary by orders of magnitudes. Similarly, also RNA half-lives in human B-cells ranged from minutes to days [10]. Some biological correlations have been identified, for example membrane proteins exhibit a significantly higher protein turnover than cytosolic proteins and phosphorylated proteins have a higher turnover than non-phosporylated proteins [11]. Yet, a compelling protein function vs. protein turnover correlation could not be established though.

During evolution, mutations to genes lead to functional divergence of their protein products but they also affect innate molecular attributes such as turnover or stability. However, if and how continuous mutagenesis changed innate molecular attributes over time is rarely discussed [12, 13]. There have been few reports of correlations between protein age and percentage of protein disorder (negative correlation) [13] or protein age and protein packing density [12]. Here, I present over a dozen additional correlations between gene age and various molecular attributes. For example, ‘old’ proteins that already existed in unicellular organisms on average have a lower turnover than ‘young’ proteins which appeared more recently. Together with other already published correlations, some also reaffirmed here, these observations suggest that there is a continuous and gradual change of different molecular attributes over time through nonsynonymous mutations. Obviously, there are countless and diverse molecular attributes such as the propensity for protein-protein-interaction [14] or the length of poly(A) tails [15]. Consequently, there is vast literature on molecular attributes and thus it is important to note that the claim here is neither to have uncovered all possible attribute-gene age correlations nor to be the first to present them specifically. Rather, the scope of this research was to show an overarching trend of attribute optimization over time by analyzing many different molecular attributes by the same, simple correlation with gene age. I find that older genes tend to produce more stable and structured proteins and mRNAs. I present a hypothesis that suggests that such optimized molecular attributes arise from cumulative mutational drifts of old genes. Consequently, young genes produce less optimized molecules. Just how mutations that favor attribute optimization tend to accumulate and become fixated is also discussed. Rather than invoking increased cellular fitness as a driving force for selection, I postulate that optimized, stable proteins and mRNA reduce the need for transcription. In turn, less transcription reduces transcription-associated mutagenesis (TAM) at this specific gene locus so that these optimizing mutations become fixated.

## Results

### Molecular stability and gene conservation correlate with gene age

Triplicate, high-throughput mass spectrometry (MS) was previously used to analyze and compare protein turnover in non-dividing arrested human cervical HeLa and differentiated mouse muscle C2C12 cells [11]. MS based on SILAC (stable isotope labeling of amino acids in cell culture) labeled amino acids can be used for quantitative protein abundance comparison between samples [16]. Similar to incorporation experiments with radioisotope-labeled amino acids decades ago [17, 18], SILAC allows the analysis of turnover of thousands of proteins. Sub-saturating metabolic incorporation of ‘heavy’ isotopes produced a turnover value (heavy / unlabeled ratio after 24 h) for 4106 human and 3574 mouse proteins [11]. Here, the half-lives of proteins and their corresponding gene age were compared to reveal if there is a general correlation between the time of existence of a gene and the stability of its protein product. Gene ages were obtained from the ProteinHistorian Database [19] and genes were taxonomically grouped as unicellular organisms (u org), unicellular eukaryotes (u euk), Ophistokonta/Bilateria/Deuterostomia (OBD), chordates (chor), or mammals (mamm). This grouping was chosen to reflect major steps in evolution. So all genes for which the protein turnover and the gene age was available were assigned to one of these five groups according to the gene age and the median protein turnover for all genes in the group was determined. It is important to note that in the figures, u org, u euk, OBD, chor, and mamm, indicate the age of the genes, not the origin. Thus, ‘chor’ genes originally appeared during the time when chordates first existed. The grouping according to gene age thus allowed comparison of human genes that already existed in prokaryotes billions of years ago with those human genes that appeared more recently and existed only since the age of mammals.

**Fig. 1:**
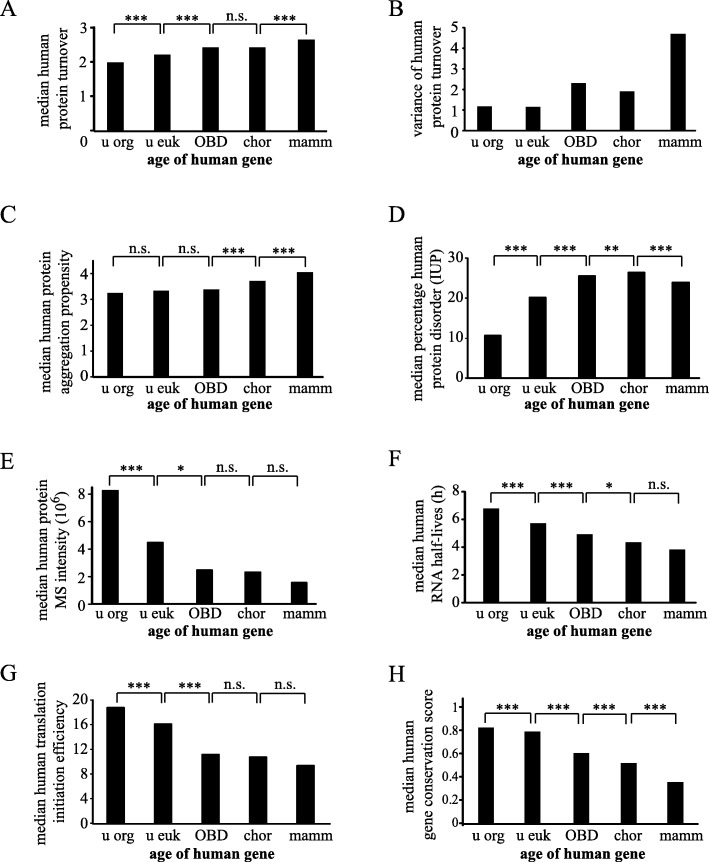
Age-dependent changes of macromolecules. **a** Median human protein turnover for taxonomic groups. Number of proteins: u org = 904, u euk = 1745, OBD = 749, chor = 378, mamm = 77. **b** Variance of median protein turnover for taxonomic groups in (**a**). **c** Median human protein aggregation propensity for taxonomic groups. Number of proteins: u org = 1842, u euk = 4005, OBD = 3745, chor = 4191, mamm = 2127. **d** Median level of protein disorder for taxonomic groups. Number of proteins: u org = 1943, u euk = 4377, OBD = 4129, chor = 4680, mamm = 2787. **e** Median protein abundance for taxonomic groups. Number of proteins: u org = 904, u euk = 1745, OBD = 749, chor = 378, mamm = 61. **f** Median mRNA half-lives for taxonomic groups. Number of mRNA species: u org = 903, u euk = 2055, OBD = 1117, chor = 538, mamm = 63 (**g**) Median human translation efficiency. Number of proteins: u org = 1219, u euk = 2948, OBD = 2039, chor = 1428, mamm = 21. **h** Median gene conservation score for taxonomic groups. Number of genes: u org = 1904, u euk = 4280, OBD = 3984, chor = 4539, mamm = 1768. (ANOVA, Bonferroni post-hoc analysis for all histograms except Fig. 1b)

For human proteins, the correlation of decreasing protein turnover with increasing protein age was significant (Spearman’s correlation, *r* = − 0.20, *P* < 0.0001) as were most differences between taxonomically grouped proteins (Fig. 1a) (ANOVA, Bonferroni post-hoc analysis; also Fig. 1c-h). A box plot of the same data is shown in Additional file 1: Figure S1a. Notably, the variance of turnover values among all proteins in each of the five groups substantially decreased with increasing protein age (Fig. 1b) indicating that turnover was more uniform for old proteins. Similarly, the protein turnover values derived from the mouse C2C12 MS experiments correlated with gene age as well (Spearman’s correlation, *r* = − 0.31, *P* < 0.0001) (Additional file 1: Figure S1b). Protein turnover of rat proteins showed the same trend (Additional file 1: Figure S1c). Analysis in *Schizosaccharomyces pombe* indicated that older genes produce proteins with longer half-lives (Additional file 1: Figure S1d). Moreover, yeast *Saccharomyces cerevisiae* proteins with prokaryotic orthologues [20] exhibited longer half-lives [2] compared to those without (52 vs. 40 min, *P* < 0.0001, Mann-Whitney test). These correlations are supported by a previous publication showing that human protein stability in terms of free energy folding ΔG was higher for old genes vs. young ones [21]. In summary, these data suggested that proteins from older genes on average exhibit a lower turnover than proteins from younger genes. To test if additional molecular attributes also follow such a trend, various other attributes were further examined.

Protein aggregation, once thought to be a characteristic of diverse diseases such as Alzheimer’s or Parkinson’s disease, is now considered to be more of a generic property of polypeptide chains [22]. I found that the overall strength of aggregation nucleating regions per protein significantly decreased with gene age, albeit weakly (Spearman’s correlation, *r* = − 0.11, *P* < 0.0001) (Fig. 1c). Additionally, it was also reported that proteins with high turnover were found to have an increased propensity to aggregate [23]. Together, this suggests that older proteins have a lower tendency to aggregate than younger ones.

Since it was demonstrated that the predicted extent of intrinsically unstructured protein (IUP) levels negatively correlated with protein half-lives [24], protein structure may itself be influenced by protein age. Indeed, the levels of unstructured regions in human proteins significantly decreased with increasing protein age (Spearman’s correlation, *r* = − 0.18, *P* < 0.0001) although there is a minor decrease rather than increase from chordates to mammals (Fig. 1d). When analyzing yeast *Saccharomyces cerevisiae* genes the same way, there was an even more pronounced correlation between protein structure and gene age (Spearman’s correlation, *r* = − 0.35, *P* < 0.0001) (Additional file 2: Figure S2a). In addition, when comparing different species, prokaryotes have been found to exhibit significantly less disordered proteins compared to eukaryotes [25, 26]. Thus, longer existing proteins are on average more structured as was independently demonstrated in a recent study [27].

Protein abundance in a data set can be approximated by summed MS peptides intensities [28, 29] as the measured peptide signal is greater for abundant proteins. In the HeLa data set, protein abundance was another molecular attribute that changed over time, as older human proteins were significantly more abundant (Spearman’s correlation *r* = 0.12, *P* < 0.0001) (Fig. 1e). Similar trends have been observed before [30] albeit often without statistics to support it. In the present study, the MS HeLa protein abundance analyses were not skewed towards high abundant proteins since abundance varied over five orders of magnitude and exhibited a bell-shaped distribution (Additional file 2: Figure S2b). Moreover, almost one hundred human transcription factors, i.e. proteins considered to be of low abundance, were detected in the HeLa lysates [11]. Similar to protein abundance, mRNA abundance was also significantly higher for old genes compared to young ones (Spearman’s correlation *r* = 0.34, *P* < 0.0001) (Additional file 2: Figure S2c).

RNA half-lives were larger for old genes compared young genes (Spearman’s correlation for human, *r* = 0.23, *P* < 0.0001; Spearman’s correlation for mouse, *r* = 0.26, *P* < 0.0001) (human in Fig. 1f, mouse in Additional file 2: Figure S2d). Analysis of RNA secondary structure in *Saccharomyces cerevisiae* genes revealed a higher level of structure, i.e. a higher average Parallel Analysis of RNA Structure (PARS) score [31], for older genes with prokaryotic orthologs (0.28 vs. 0.23, *P* < 0.0001, Mann-Whitney test) compared to younger genes without orthologs.

The translation initiation efficiency describes how well a particular mRNA assembles with 80S ribosomes which is an indication of the fraction of mRNA molecules that can be translated. Genes with higher values are thus able to produce more proteins within a certain time window. Using published data on the translation initiation efficiency in human HeLa cells [32] older genes on average were found to have higher translation efficiencies than younger ones (Fig. 1g) (Spearman’s correlation *r* = 0.08, *P* < 0.0001).

Taken together, the data demonstrate in different organisms that newly evolved genes tend to exhibit molecular attributes that are not well optimized, such as less stability and less structure of RNAs and proteins. In addition, the human gene conservation score [33] was as expected significantly lower in young compared to old genes (Spearman’s correlation, *r* = 0.67, *P* < 0.0001) (Fig. 1h). When analyzing more defined gene age classes (Additional file 2: Figure S2e), this correlation with conservation becomes even more obvious with an almost steady decrease with decreasing gene age, an observation that was not reported in previous publications [34].

### Continued mutagenesis decreases individual protein turnover variance

**Fig. 2:**
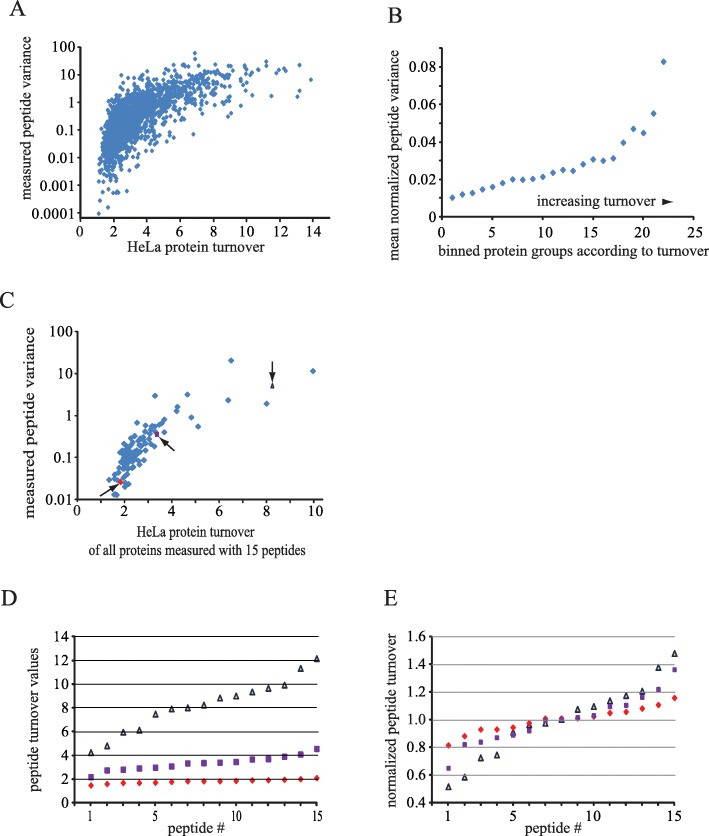
Turnover-dependent decrease in peptide variance. **a** Variance of all HeLa peptides used to compute individual protein-specific turnover values. **b** Median of normalized peptide variance (Variance/(median turnover ratio)^2^) plotted in bins of proteins sorted according to their median turnover derived from all measured peptides. **c** Peptide variance of all proteins that were measured with exactly 15 peptides. **d** Measured peptide values for three proteins indicated with arrows in (**c**). Scavenger mRNA-decapping enzyme DcpS (), Tight junction protein ZO-1 (), Ferritin heavy chain () (**e**) Normalization of (**d**) by division through the median protein turnover value of the respective protein

Figure 1b demonstrated that the spread, i.e. variance of human turnover values of all proteins in a respective taxonomic group was highest for young proteins. The question then arose if the spread of measured turnover values for each specific protein also changed with age. In SILAC-MS experiments [35], the various peptides that identify a specific protein are often quantified multiple times and so one can determine the variance of the measured turnover values for each protein separately. Indeed, the protein-specific variance was highest for high turnover proteins and thus the variance indirectly related to gene age. Plotting the variance of measured peptide values versus protein turnover shows (Fig. 2a,b) that the peptide value variance was much greater for high turnover compared to low turnover proteins. These data therefore suggest that for a few low turnover proteins, the variance can decrease to a level of near uniformity. For display purposes, all proteins whose overall turnover value happened to be based on 15 peptide measurements are shown in Fig. 2c and the spread of variances recapitulates that pattern seen in Fig. 2a. The variance of three exemplary proteins (Fig. 2d) and normalization of the respective peptide values confirmed the trend (Fig. 2e) of low turnover proteins exhibiting much less variance. Therefore, each individual molecule of an old protein exists more or less the same length of time before it gets degraded. Conversely, for a protein of a younger gene, the time it takes to be degraded following its synthesis can vary substantially.

### Molecular attributes change independently of function and essentiality

It has long been assumed that core cellular proteins (‘housekeeping genes’) are ubiquitously expressed and intuitively should be more conserved [36]. However, from young to old human housekeeping genes [37], not only did I observe a continuous increase in gene conservation, but also in protein structure as well as mRNA and protein turnover (Additional file 3: Figure S3a,b,c,d). The family of human transcription factors [38] exhibited the same trend (Additional file 4: Figure S4a,b,c,d). Both sets of data thus suggest that the optimization of molecular attributes over time occurs independently of essentiality and function. Also, the mean conservation score of human genes (0.71) considered to be essential [39] was significantly lower than the mean of those genes that first appeared in prokaryotes (0.82; *P* < 0.0001, Mann-Whitney test) or unicellular eukaryotes (0.78; *P* = 0.0002, Mann-Whitney test). Thus, old genes are more conserved than essential genes and consequently protein function appears to have lesser role in determining gene conservation than generally assumed. In addition, essential mouse genes [40] also showed an increase towards higher mRNA and protein stability from young genes to old ones (Additional file 4: Figure S4e,f).

### GO categories

GO (gene ontology) biological process analyses of human and mouse turnover data allowed ranking of GO categories according to their median turnover values (Additional file 6: Tables S1,S2). In the present HeLa and C2C12 data sets, core cellular and metabolic categories that must have appeared early in evolution such as ‘protein synthesis’ displayed low median turnover values while categories central to the appearance of multi-cellular organisms and vertebrates exhibited high and even higher values, respectively. Analogous trends were previously also seen in other analyses such as GO categorization of protein conservation scores [41] or RNA half-lives [10]. Comparing the order of almost 600 GO categories between mouse and human indicated a high similarity (Spearman’s correlation, *r* = 0.67, *P* < 0.0001). However, because of extensive cross-annotations and functional overlap between categories, it was not possible to establish a common temporal ranking based on both species. Instead, a manually curated selection of human categories is shown in Table 1. The data (Additional file 6: Tables S1,S2) suggest that one of the first biological processes to exist was ATP production. Other basic biological processes such as translation or biosynthesis of carbohydrates also exhibited low average turnover values while biological processes related to complex multicellular organisms such as embryonic morphogenesis or axon guidance had significantly higher values. Undoubtedly, this ranking provides an appealing ‘time stamp’ for the time of emergence of biological processes although this ranking must be considered preliminary. Such an approach can clearly not be extended to individual proteins as the turnover based GO ranking only became meaningful if several proteins were included. Nevertheless, one can approximate the coarse order of many of these processes based on known cellular physiology. For example, nuclear transport as GO category must have appeared in eukaryotes not prokaryotes, while the GO categories translation and carbohydrate metabolism must have appeared in prokaryotes. Similarly, GO categories such as cell motion or response to chemical stimulus are relevant to unicellular organisms and thus appeared early in evolution while GO categories such as organ or nervous system development are key features of multi-cellular organisms and therefore must have appeared later. In conclusion, the GO data independently support the gene age – protein turnover correlation.

**Table 1:** Ranking GO categories of biological processes according to their mean protein turnover. Shown is a manually curated list of GO categories following a GO category analysis of HeLa proteins of the MS data set. Categories whose median turnover was significantly different from the overall HeLa turnover data set value (2.2) are indicated in the right column

GO Category	Biological Process	Mean Turnover	# of Proteins	Difference to total Protein Turnover
GO:0006412	translation	1.82	229	***
GO:0006520	cellular amino acid metabolic process	1.89	110	***
GO:0006807	nitrogen compound metabolic process	1.89	142	***
GO:0044262	cellular carbohydrate metabolic process	1.91	108	***
GO:0006631	fatty acid metabolic process	1.92	63	*
GO:0009117	nucleotide metabolic process	1.93	84	***
GO:0005975	carbohydrate metabolic process	1.94	141	***
GO:0006066	alcohol metabolic process	1.95	107	*
GO:0051186	cofactor metabolic process	1.99	89	**
GO:0006091	generation of precursor metabolites and energy	2.03	131	*
GO:0042221	response to chemical stimulus	2.06	152	
GO:0006457	protein folding	2.07	109	
GO:0000398	nuclear mRNA splicing, via spliceosome	2.08	105	
GO:0006928	cell motion	2.09	85	
GO:0051169	nuclear transport	2.11	62	
GO:0006811	ion transport	2.12	100	
GO:0042981	regulation of apoptosis	2.12	185	
GO:0006396	RNA processing	2.17	278	
GO:0007600	sensory perception	2.19	50	
GO:0050793	regulation of developmental process	2.21	247	
GO:0007399	nervous system development	2.25	54	
GO:0044419	interspecies interaction between organisms	2.26	119	
GO:0051704	multi-organism process	2.29	140	
GO:0006955	immune response	2.31	73	
GO:0007165	signal transduction	2.31	556	***
GO:0006281	DNA repair	2.32	106	*
GO:0009653	anatomical structure morphogenesis	2.38	131	**
GO:0010324	membrane invagination	2.39	65	***
GO:0010468	regulation of gene expression	2.40	503	***
GO:0008202	steroid metabolic process	2.48	57	***
GO:0007155	cell adhesion	2.50	116	***
GO:0007166	cell surface receptor linked signal transduction	2.51	151	**
GO:0032774	RNA biosynthetic process	2.51	72	***
GO:0048513	organ development	2.52	126	***
GO:0007275	multicellular organismal development	2.64	111	***
GO:0051301	cell division	2.65	108	***

***P < 0.001; **P < 0.01; *P < 0.05

### A hypothesis for dynamic molecular attribute optimization

**Fig. 3:**
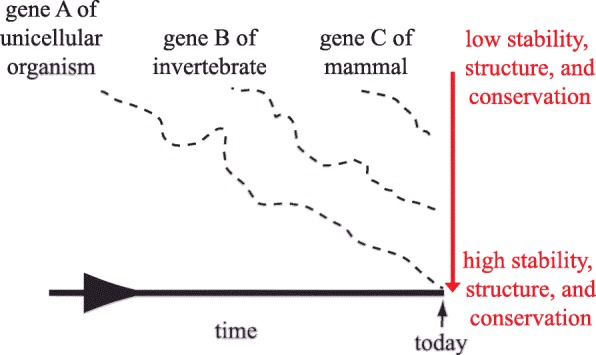
Hypothesis: Dynamic evolution of molecular attributes. Time increases from left to right and molecular optimization increases from top to bottom. As time progresses, macromolecular optimization continuously increases, so that longer existing macromolecules are generally more optimized. Consequently, longer existing macromolecules exhibit more structure and more stability

The data discussed above showed that in different organisms, attributes such as half-life of proteins and RNAs significantly change over time. This temporal correlation can be interpreted in two ways. First, the molecular attributes of molecules that appeared billions of years ago are very different from those that arose only millions of years ago. This implies creation of stable, structured proteins with long mRNA half-lives in prokaryotes, but less stable and less structured proteins with shorter mRNA half-lives in mammals, and proteins with intermediate qualities in between. This broadly relates to the ‘constant restraint’ model [34, 42]. A second, alternative explanation would be that newly evolved genes, from the time of prokaryotic life to the age of mammals, always have the tendency to produce more unstructured and unstable molecules when they first appear. Continued mutagenesis then gradually optimizes the respective molecular attributes over time so that the oldest, prokaryotic genes were subjected to the most extensive optimization and thus produce the most structured proteins as well as the most stable mRNAs and proteins today. Genes that appeared later when multicellular organisms and invertebrates first inhabited Earth had less time available and therefore were less optimized compared to prokaryotic genes, but are more optimized compared to those that arose in mammals. Genes that appeared the latest at the age of mammals had the least time available for optimization and thus produce the least structured and least stable proteins and mRNAs. This hypothesis is graphically visualized in Fig. 3. Genes A, B, and C represent genes that first appeared in prokaryotes, multicellular organisms/invertebrates, or mammals, respectively, and so gene A is older than B, and B is older than C. The vertical axis indicates the degree of molecular stability, structure, and gene conservation - unstructured, unstable molecules from less conserved genes are at the top while structured, stable molecules from conserved genes are at the bottom of the axis. The stippled lines reflect the path to greater optimization of each gene over time. ‘Newborn’, imperfect genes generally start their path near the top of the axis and then gradually, but not steadily, their molecular attributes change to reach the bottom of the graph. The hypothesis thus proposes that proteins and mRNAs which were present in the first forms of unicellular life were optimized subsequently during the billions of years until today to now have a low uniform turnover and high conservation. Molecules that appeared later in evolution also change but are more ‘work in progress’ and will, like all other proteins and mRNAs, continue to be optimized. Consequently, the wide spectra of half-lives, protein disorder, and degrees of conservation we currently observe actually represent a snapshot of molecules being at different stages along their evolutionary paths.

### Inter-species differences

**Fig. 4:**
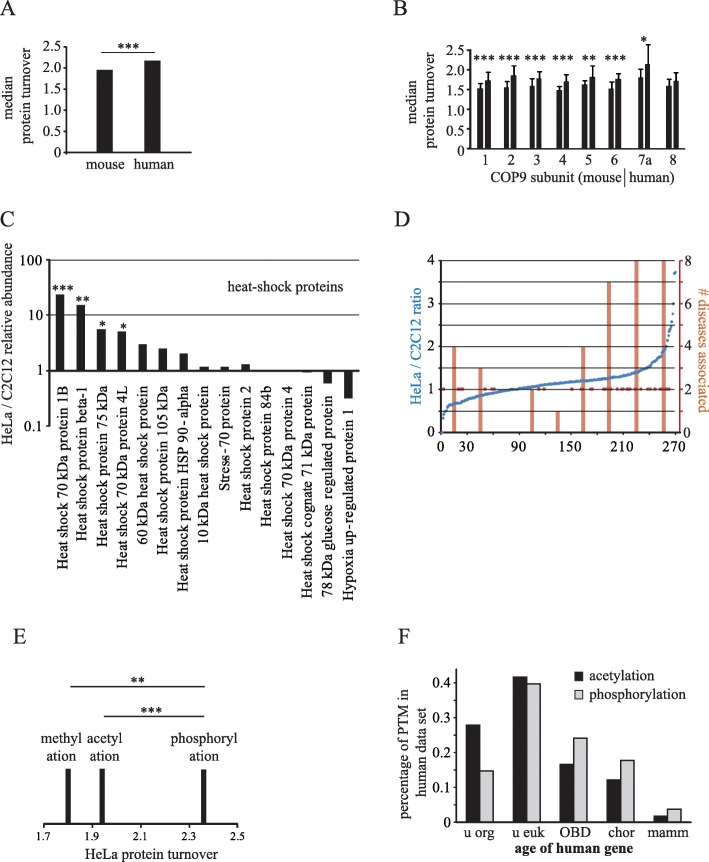
Age-dependent inter-species differences and post-translational modifications. **a** Median protein turnover of 2107 mouse and human homologs. **b** Median peptide turnover values of COP9 signaling complex proteins in mouse and human. **c** Relative human vs. mouse abundance of heat-shock proteins as determined by mass spectrometry summed peptide peaks. **d** Incidence of human diseases vs. ratio of human/mouse protein turnover. Blue dots: distribution of ratios of 269 orthologs. Brown dots: distribution of diseases. Brown bars: sum of diseases for bins of 30 proteins. **e** Median distribution of post-translational modifications in the HeLa data set. Only proteins were included that exclusively have one of the PTMs but not the others: meth. = 27, acet. = 413, phos. = 1497. **f** Relative fractions of acetylation (933 proteins) and phosphorylation (2699 proteins) in human HeLa data sets

So far, all the presented data were based on analyses of proteins or RNAs *within* one species with the observation that longer existing genes and their products were different to younger ones. Because of their longer existence, older genes have been subject to more mutagenesis over time and the extent of mutagenesis a gene experienced determines the extent of optimization of molecular attributes. Consequently, within one species, the products of older genes are more stable and more structured. Overall, when attribute data from two separate species could be obtained, consistently I found a larger age-attribute correlation value for those species that have a higher cumulative mutational load. For example, when comparing yeast with human proteins, yeast proteins displayed a higher correlation between gene age and unstructured protein regions. Also, there was a higher correlation for gene age and RNA turnover in mice than men. Thus, the prediction would be that proteins from species with higher mutational rates are more optimized. Comparing protein turnover in mammalian cells vs. lower organisms would be difficult as metabolic rates, cell cycles, or body temperatures can be very different. Therefore, protein turnover was compared in human vs. mouse using the two aforementioned data sets from non-dividing HeLa and C2C12 cells [11]. Mice have higher mutational rates [43] and therefore the expectation would be that the average protein turnover is decreased compared to human. The mean turnover value in human was 2.2 and in mice 2.0 (*P* < 0.0001, Mann-Whitney test) (Fig. 4a). Most (73%) of the 2107 protein homologs identified in both species exhibited a larger value in humans. The same trend was seen in individual subunits of several previously characterized protein complexes such as the COP9 complex (Fig. 4b, Additional file 5: Table S3, Figure S5a,b). Also, the fraction of human proteins with lower turnover values in mice was increasing with decreasing turnover values (Additional file 5: Figure S5c). Thus, the lower the human turnover value, the higher the probability that the corresponding mouse homolog displayed an even smaller value. The data therefore support the idea that increased mutagenesis leads to more optimized proteins as mouse orthologs exhibited on average lower turnover compared to human. If this were the case, one would expect that less optimized human proteins required more ‘cellular support’ to maintain proper functioning. Relative quantitative proteome comparison of mouse and human data showed that heat shock proteins were much more abundant in human compared to mouse cells (Fig. 4c). This is in line with the heat shock capacitor hypothesis that views molecular chaperones as key mediators of adaptive evolution by buffering genetic variation [44]. Of course, although the data showed highly significant trends that were as predicted, further analyses are certainly necessary to confirm the results of such inter-species comparisons. Nevertheless, data from both, intra-species and inter-species comparisons suggest that the cumulative levels of mutagenesis affect molecular attributes.

### Protein turnover and cellular fitness

To assess if high turnover proteins affect cellular fitness, the protein turnover values of all genes in the ENSEMBL database known to produce lethal phenotypes were correlated. Such an unbiased approach did not reveal any obvious association between known lethal diseases and human proteins of high turnover. However, there was a clear association between lethal phenotypes and the ratio of human/mouse turnover values from orthologs detected in both species. As expected from the high correlation of turnover values between both species [11], most orthologs displayed a human/mouse turnover ratio close to 1 (blue dots, Fig. 4d). Bins of proteins (30 each) with a ratio close to 1 (bins 31–60, 61–90, and 91–120) had a total of zero, one, two, or three lethal diseases associated (brown bars and dots). But for orthologs with very high or low ratios however, there were up to eight counts of diseases. These extreme ratios and thus extreme differences between human and mouse turnover values of the same protein may be an indication that these proteins are particularly prone to mutagenesis and thus one could speculate that they have a higher chance to acquire lethal phenotypes.

### Continued mutagenesis and post-translational modifications

We previously showed that phosphorylated proteins have a significantly higher turnover than non-phosphorylated proteins [11]. I now find that acetylated [45] and methylated [46] proteins have a significantly lower turnover than phosphorylated proteins (Fig. 4e). Since the data presented here suggest that continued mutagenesis decreases protein turnover, this mutagenesis may also influence the type of post-translational modifications (PTMs). The abundance of post-translational modifications in humans is phosphorylation > > acetylation > methylation [45, 47, 48]. This is opposite to prokaryotes such as *Escherichia coli* where the abundance of phosphorylation < acetylation [49, 50] or the spirochete *Leptospira interrogans* where phosphorylation < acetylation < methylation [51]. Thus, prokaryotes tend to have proteins that are methylated and acetylated while phosphorylation is predominant in humans. Similar to other molecular attributes, PTMs could potentially correlate with gene age as well. Analysis of PTMs in human proteins showed that acetylation was significantly more associated with older proteins than phosphorylation (Mann-Whitney test, *P* < 0.0001). For example, for very old proteins that originated during the stage of ‘unicellular organisms’, the percentage of acetylated proteins was twice as high compared to the percentage of phosphorylated proteins (Fig. 4f). Given that young proteins tend to be phosphorylated and older proteins are more likely to be acetylated, I would like to speculate that post-translational modifications are also molecular attributes that are undergoing ‘optimization’ because of continued mutagenesis. Notably, phosphorylation was found to occur mostly in unstructured regions of proteins [52] furthering the notion that younger genes produce proteins with less structure and more phosphorylation. The analyses presented here thus suggest that the existence of PTMs on proteins is ultimately transient as proteins become older. As the hydrophobicity of the actual PTM increases from phosphorylation to acetylation and then methylation, water solubility and protein-protein interactions should be affected. According to the hypothesis, proteins are therefore more likely to be phosphorylated at ‘birth’, yet they eventually lose this modification and may acquire an acetylation or methylation subsequently as mutations optimize the proteins towards higher stability. However, it is not clear at this point whether changes in PTMs relate at all to changes protein turnover. Turnover and PTMs may simply be independent manifestations of continued mutagenesis. Also, whether the changing PTMs locate to the same or different positions within the protein will have to be determined.

## Discussion

Here, I presented data which show that molecules have distinct properties according to their time of birth independent of function, essentiality, or expression. Unless one were to postulate that nature ‘by design’ progressively produces increasingly unstructured and unstable proteins and RNAs, the data are best accommodated by the proposed hypothesis assuming that new genes are imperfect at the beginning and are then subjected to optimization over time. At least in respect to protein turnover, this optimization also reduces variance. The surprisingly smooth, almost steady decrease of conservation for genes that appeared later in evolution (Additional file 2: Figure S2e) is an appealing representation of such optimization. Importantly, the hypothesis does not require that the different attributes of a specific molecule must correlate. In other words, a gene that produces a stable mRNA may in turn give rise to a protein with high turnover because a mutation that changes mRNA stability may have little effect on protein turnover or vice versa. Indeed, published data showed that protein and mRNA half-lives did not correlate [53]. Moreover, the hypothesis allows for genetic selection to maintain high turnover or less structure where functionally required. For example, proteins that functionally require short half-lives such as cyclines possibly optimize only mRNA half-lives and protein structure, independent of a selective pressure that supports high protein turnover.

Protein abundance also correlated with gene age and molecular optimization may indeed increase abundance over time, similar to structure und half-lives (Fig. 1a,d). However, a different interpretation of the data would be that the increased protein abundance is a direct consequence of increased protein structure, increased protein half-lives, and increased mRNA half-lives. Especially changes in mRNA and protein stability should lead to accumulation of protein and both are expected to act independently of each other [53]. Not surprisingly, the abundance of mRNAs was also higher for old genes compared to young ones.

The observed gradual change of attributes could be the consequence of two opposing scenarios. These scenarios are highlighted below using the degree of structure of transcription factors for illustration. In one scenario, nature produced highly structured transcription factors billions of years ago but created transcription factors with much more disorder millions of years ago, and transcription factors with intermediate structure in between. Thus, the molecular attributes of transcription factors were very different depending on the time when they were ‘born’. This appears to be prevailing view of molecular attribute genesis, but an understanding of the molecular basis and the driving force behind this scenario is vague at best [27]. It is difficult to imagine that protein function is the underlying driving force responsible for producing less and less structured transcription factors over time when the principles of transcription are the same for all transcription factors, young and old. If having less structure were beneficial, why did nature only produce such transcription factors millions, but not billions of years ago?

Conversely, a second, more compelling scenario to explain the correlation of molecular attributes with gene age is the following: Rather than assuming that newly existing molecules of recent times have different attributes than those that appeared long time ago, I propose a hypothesis that posits that new genes always, from billions of years ago to millions years ago, had imperfect attributes at the beginning of their existence which were then optimized through mutagenesis over time. This implies that a transcription factor that was present billions of years ago also exhibited less protein structure billions of years ago, but nature optimized its various attributes so that the *same* transcription factor today is highly structured. I also described the correlation of several other molecular attributes with gene age, including half-lives of proteins and RNAs. Together, all these observations suggest that mutations not only change the function and activity of a molecule but also dynamically and continuously change its innate attributes.

How are the different optimized attributes fixated and selected for during evolution? For example, reduced turnover requires less energy for cellular homeostasis, but these changes are incremental as the energy needs for the production of one protein compared to all proteins in a cell is negligible. With more than 10,000 different protein species detected in eukaryotic cells [54], changing the turnover of a single specific protein will correspondingly affect about 1/10000 of the energy used for protein production. The degree to which this confers a competitive growth advantage is not clear. The cellular consequences of stepwise optimizing any of the other attributes also appear negligible. I therefore wish to propose an alternative explanation. Hypothetically, if an mRNA species becomes ten times more stable, ten times less transcription should be necessary to maintain equal levels of steady-state mRNA abundance. I showed here that old genes produce more stable mRNAs and proteins, and so their individual transcription rates should be reduced. Use-dependent transcription-associated mutagenesis (TAM) of genomic DNA [55] is a possible link between optimization, transcription rates, and fixation of mutations. TAM is locally altering and destabilizing the genomic DNA template through a variety of different mechanisms [56]. Thus, instead of invoking selective pressure for such mutations that optimize molecular attributes such as protein turnover, perhaps these mutations become fixated because they directly reduce mutagenesis at their own gene locus. If a random mutation produces more stable mRNA or protein, less transcription should be necessary to reach normal levels of abundance and consequently, fixation of this gene increases as it is more protected from mutations because of reduced TAM. Indeed, I found a significant negative correlation between gene conservation scores and corresponding transcriptional rates [10] (Spearman’s correlation, *r* = − 0.18, *P* < 0.0001) which suggests that reduced transcription accounts to some extent for the increased fixation of old genes. From a molecular perspective, any random mutation that reduces the necessity for transcription, i.e. by ultimately increasing mRNA and protein abundance, should principally be fixated because of TAM. Such fixation would occur ‘passively’ and not by selection based on function. To this end, it is known that old, conserved housekeeping genes generally exhibit lower evolutionary rates [57]. Since I showed here that old genes have on average more stable mRNAs and proteins, the reduced TAM should be in line with their lower evolutionary rates.

## Conclusions

Molecular attributes such as protein or RNA stability often vary over several magnitudes but a conclusive explanation for this variability has been missing. A new hypothesis was introduced that views individual attributes of every molecule as being at different stages along a path of optimization based on continued mutagenesis over time. The hypothesis does not attempt to link the various attributes of the same molecule with function but rather allows attributes to be affected independently by a mutation. Thus, the biological challenge would be to maintain molecular function in the face of ever changing molecular attributes. Based on these assumptions, this hypothesis can be tested by in vitro evolution.

## Methods

All analyses were based on previously published data. Statistical data analyses were performed using Prism 5.0 (GraphPad Software, San Diego, CA USA). Each gene obtained from the different molecular attributes data sets was given its published age as described in the database for human gene/protein age (‘ProteinHistorian’/ http://lighthouse.ucsf.edu/ProteinHistorian/) [19]. According to this age, genes in each data set were grouped. Each taxonomic grouping of the ProteinHistorian output is indicated in Additional file 2: Figure S2e, together with the corresponding gene ages.

Protein and peptide turnover values as well as protein abundance were from a previous publication [11]. RNA folding energies (PARS) [31] (http://genie.weizmann.ac.il/pubs/PARS10/pars10_catalogs.html), gene conservation scores (https://dsgweb.wustl.edu/hutz/index.html), and bacterial orthologues in yeast (http://makana.berkeley.edu/phylofacts/) [20] were obtained from publicly available databases. RNA half-lives were previously published [10] from which also the relative transcription rates were derived assuming that transcription rates are proportional to degradation rates at steady state. Yeast protein half-lives [2], human transcription factors [38], human housekeeping genes [57], human [39] and mouse [40] essential genes, acetylated [45] and phosphorylated [47] proteins, human protein aggregation propensity [58], human translation initiation values [32], human mRNA abundance [59] were each obtained from the indicated references. The mean normalized peptide variance was defined as (mean variance) / (mean turnover value)^2^ for all proteins in the respective bin (93 proteins/bin). Peptides were required to be quantified with at least three peptides during the MS experiments.

Ranking of biological process GO categories was based on the median protein turnover of each particular category. In parallel, 1000 sets of random protein turnover values were iteratively created (“bootstrapping”) where each set contained as many values as the number in the respective category and the mean of all 1000 random sets produced the bootstrap distribution. The difference between the bootstrap distribution and the mean of the category was computed in standard deviations and yielded a probability value for a null correlation [11]. An unbiased description of diseases associated with human proteins was obtained from the biomart database ‘MIM Morbid’ (www.ensemble.org). The MaxQuant software [60] was used for label-free quantitative analysis [61] of mouse and human proteomes [11].

## Supplementary information

**Additional file 1: Figure S1.** Age-dependent changes of protein turnover. (a) Box plot of human protein turnover (Fig. 1a). (b) Median mouse protein turnover for taxonomic groups. Number of proteins: u org=751, u euk=1331, OBD=667, chor=273, mamm=46. (c) Median rat protein degradation rates for taxonomic groups. Number of proteins: u org=188, u euk=461, OBD=558, chor=439, mamm=139. (d) Mean Schizosaccharomyces pombe protein half-lives for taxonomic groups. Number of proteins: u org=409, u euk=915, Ophistokonta (Oph)=407, Ascomycota (Asc)=484, Sch.Po.=737.

**Additional file 2: Figure S2.** Age-dependent changes of macromolecules. (a) Median level of protein disorder for taxonomic groups in yeast. Number of proteins: u org=1336, u euk=1693, OBD=562, chor=1192, mamm=1070. (b) Distribution of protein abundance of HeLa MS data set. (c) Median human mRNA abundance for taxonomic groups. u org=190, u euk=337, OBD=208, chor=163, mamm=51. (d) Median mouse mRNA half-lives for taxonomic groups. Number of different mRNA species: u org=1072, u euk=2553, OBD=1636, chor=1122, mamm=313. (e) Mean gene conservation score for taxonomic groups. This graph is based on the same data as Fig. 1h but without the grouping to more general taxonomies. The number of genes is given in parentheses (ANOVA, Bonferroni post-hoc analysis for Suppl. Fig. 1 a,c,d,e).

**Additional file 3: Figure S3.** Age-dependent changes of human housekeeping genes. (a) Mean gene conservation score for taxonomic groups. Number of genes: u org=394, u euk=852, OBD=369, chor=237, mamm=39. (b) Median level of protein disorder for taxonomic groups. Number of proteins: u org=385, u euk=823, OBD=366, chor=225, mamm=38. (c) Median protein turnover for taxonomic groups. Number of proteins: u org=303, u euk=588, OBD=167, chor=85, mamm=10. (d) Median mRNA half-lives for taxonomic groups. Number of different mRNA species: u org=360, u euk=793, OBD=341, chor=220, mamm=40. (ANOVA, Bonferroni post-hoc analysis for all figures).

**Additional file 4: Figure S4**. Age-dependent changes of human transcription factors and mouse essential genes. (a) Median protein turnover for taxonomic groups of transcription factors. Number of proteins: u org+ u euk=34, OBD=34, chor+ mamm=23. Groups with less than 10 genes were added to the neighboring group (also in b,c,d) (b) Median mRNA half-lives for taxonomic groups of transcription factors. Number of different mRNA species: u org+ u euk=51, OBD=172, chor=175, mamm=89.(c) Mean gene conservation score for taxonomic groups of transcription factors. Number of genes: u org+u euk=79, OBD=471, chor=509, mamm=241. (d) Median level of protein disorder for taxonomic groups of transcription factors. Number of proteins: prok+ u euk=82, OBD=531, chor=512, mamm=322. (e) Median protein turnover for taxonomic groups of mouse essential genes. Number of proteins: u org=92, u euk=168, OBD=129, chor+ mamm=73. (f) Median mRNA half-lives for taxonomic groups of mouse essential genes. Number of different mRNA species: u org=133, u euk=296, OBD=327, chor=222, mamm=13. (ANOVA, Bonferroni post-hoc analysis for all figures).

**Additional file 5: Table S3** and **Figure S5. Table S3:** Median protein turnover values for GO categories based on mass spectrometry proteomics data from arrested human HeLa and differentiated mouse muscle C2C12 cells. Probability indicates difference to average turnover of all proteins. **Figure S5:** Evolutionary changes of protein turnover between human and mouse. (a) Graphical representation of Supplementary Table 3. (b) Median peptide turnover values of the ‘histone deacetylase and nucleosome remodeling activities complex’ proteins. (c) Grouping of proteins into equal size bins of 210 and quantification of homologous proteins with a turnover increase from human to mouse.

**Additional file 6: Tables S1** and **S2.** Median protein turnover values for GO categories based on mass spectrometry proteomics data from arrested human HeLa and differentiated mouse muscle C2C12 cells. Probability indicates difference to average turnover of all proteins.

## Data Availability

For every figure, all source data have been published before and can be obtained from the indicated publications. Processed source data that support the findings of this study are available from the corresponding author on reasonable request.

## Abbreviations

GO: Gene ontology
IUP: Intrinsically unstructured protein
MS: Mass spectrometry
PTM: Post-translational modifications
SILAC: Stable isotope labeling of amino acids in cell culture
TAM: Transcription-associated mutagenesis
