# Supplementary figures and images for: Hypothesis: protein and RNA attributes are continuously optimized over time
Source: BMC Genomics. 2019 Dec 23;20:1012. doi: 10.1186/s12864-019-6371-0 (PMC6929361; doi:10.1186/s12864-019-6371-0)

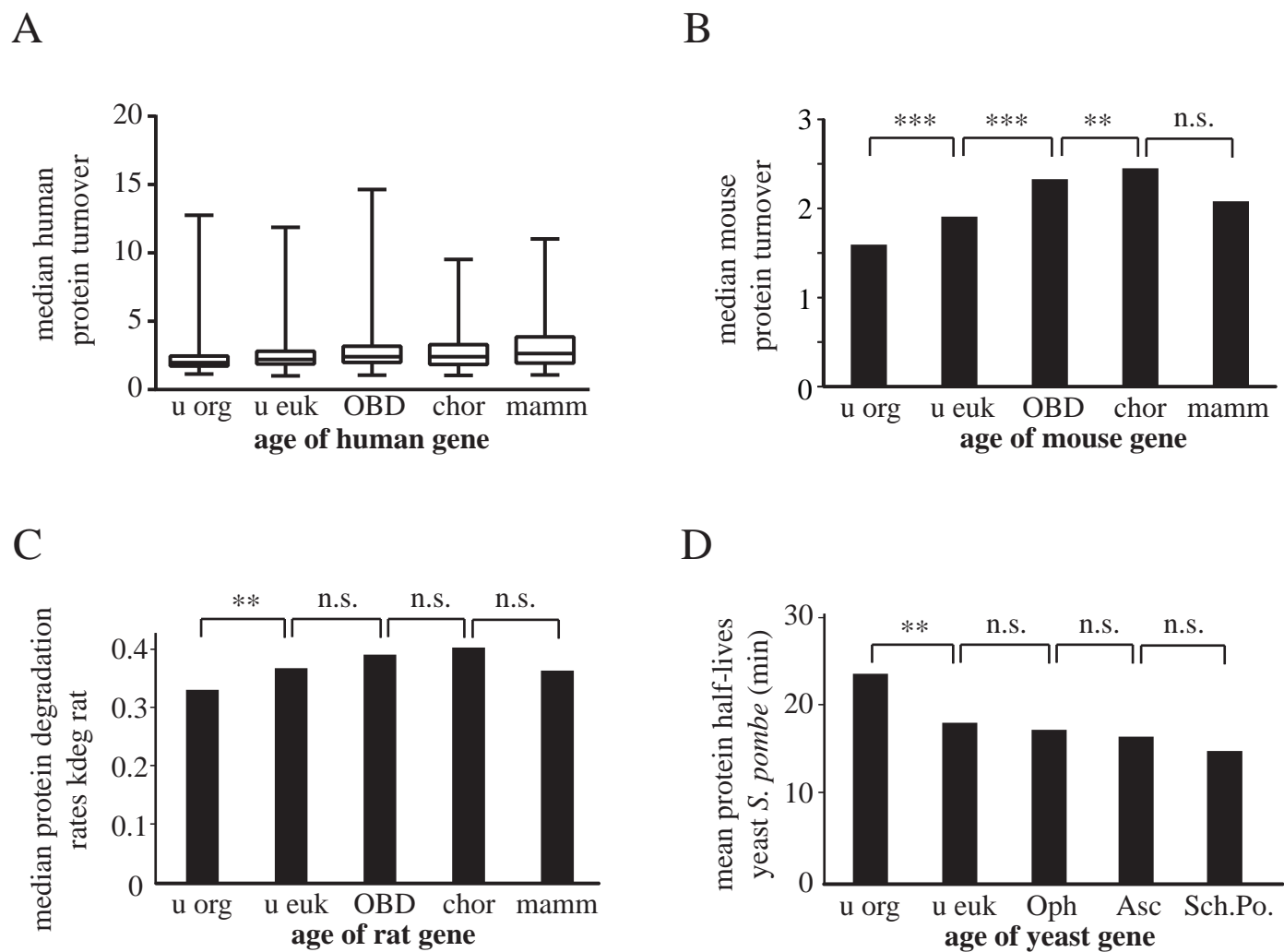

Supplementary Figure 1

Supplement: Supplementary file 1 — Additional file 1: Figure S1. Age-dependent changes of protein turnover. (a) Box plot of human protein turnover (Fig. 1a). (b) Median mouse protein turnover for taxonomic groups. Number of proteins: u org=751, u euk=1331, OBD=667, chor=273, mamm=46. (c) Median rat protein degradation rates for taxonomic groups. Number of proteins: u org=188, u euk=461, OBD=558, chor=439, mamm=139. (d) Mean Schizosaccharomyces pombe protein half-lives for taxonomic groups. Number of proteins: u org=409, u euk=915, Ophistokonta (Oph)=407, Ascomycota (Asc)=484, Sch.Po.=737. [file 12864_2019_6371_MOESM1_ESM.pdf]

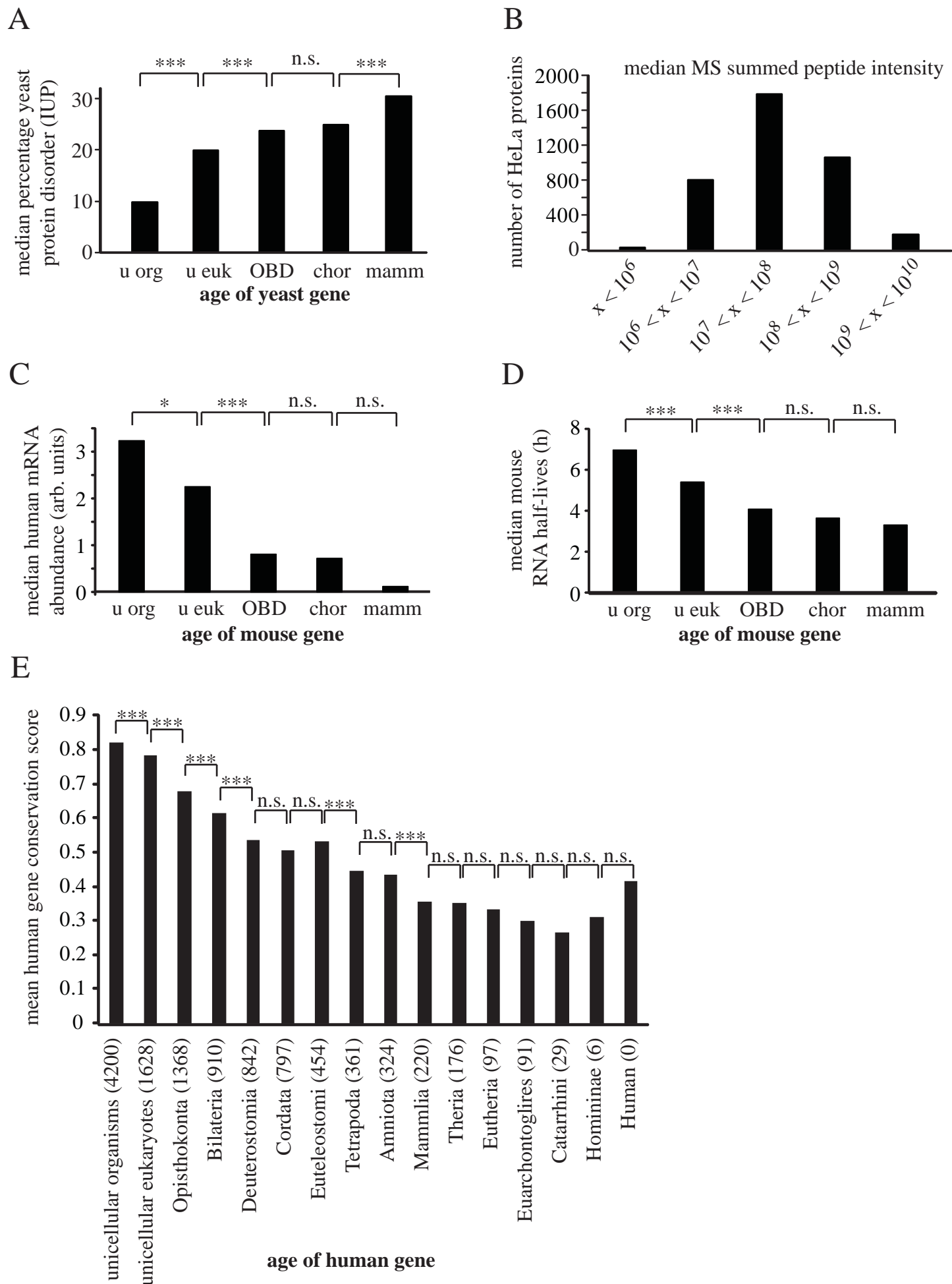

Supplementary Figure 2

Supplement: Supplementary file 2 — Additional file 2: Figure S2. Age-dependent changes of macromolecules. (a) Median level of protein disorder for taxonomic groups in yeast. Number of proteins: u org=1336, u euk=1693, OBD=562, chor=1192, mamm=1070. (b) Distribution of protein abundance of HeLa MS data set. (c) Median human mRNA abundance for taxonomic groups. u org=190, u euk=337, OBD=208, chor=163, mamm=51. (d) Median mouse mRNA half-lives for taxonomic groups. Number of different mRNA species: u org=1072, u euk=2553, OBD=1636, chor=1122, mamm=313. (e) Mean gene conservation score for taxonomic groups. This graph is based on the same data as Fig. 1h but without the grouping to more general taxonomies. The number of genes is given in parentheses (ANOVA, Bonferroni post-hoc analysis for Suppl. Fig. 1 a,c,d,e). [file 12864_2019_6371_MOESM2_ESM.pdf]

A

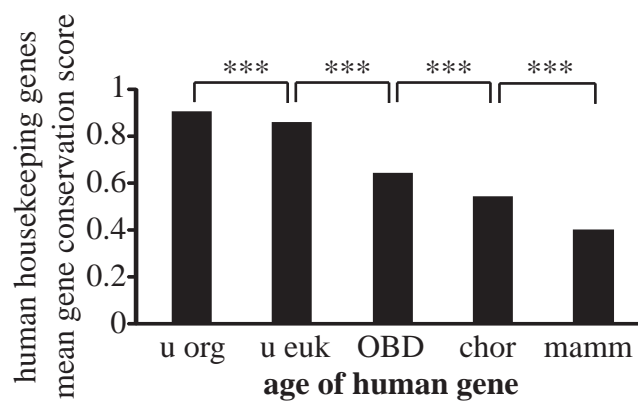

B

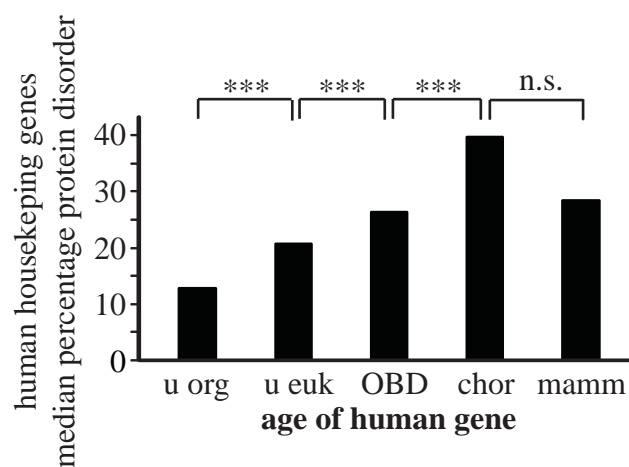

C

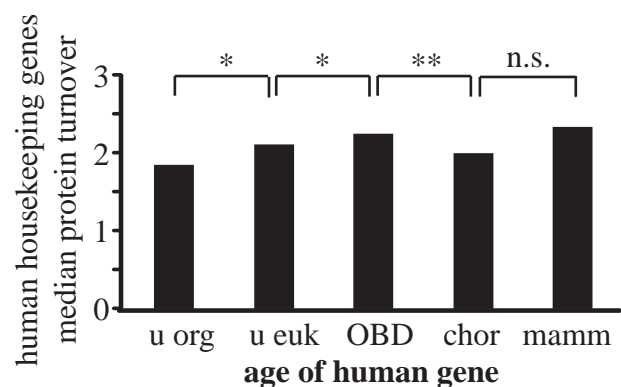

D

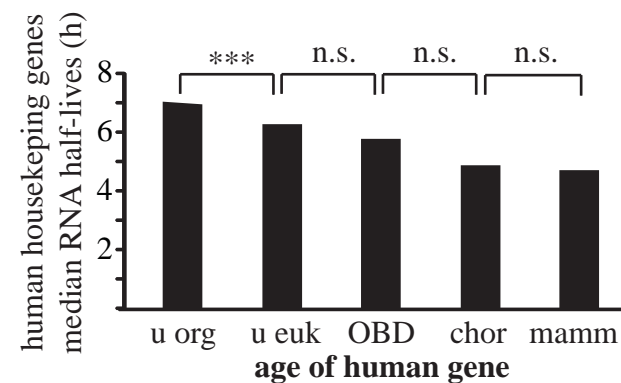

Supplement: Supplementary file 3 — Additional file 3: Figure S3. Age-dependent changes of human housekeeping genes. (a) Mean gene conservation score for taxonomic groups. Number of genes: u org=394, u euk=852, OBD=369, chor=237, mamm=39. (b) Median level of protein disorder for taxonomic groups. Number of proteins: u org=385, u euk=823, OBD=366, chor=225, mamm=38. (c) Median protein turnover for taxonomic groups. Number of proteins: u org=303, u euk=588, OBD=167, chor=85, mamm=10. (d) Median mRNA half-lives for taxonomic groups. Number of different mRNA species: u org=360, u euk=793, OBD=341, chor=220, mamm=40. (ANOVA, Bonferroni post-hoc analysis for all figures). [file 12864_2019_6371_MOESM3_ESM.pdf]

A

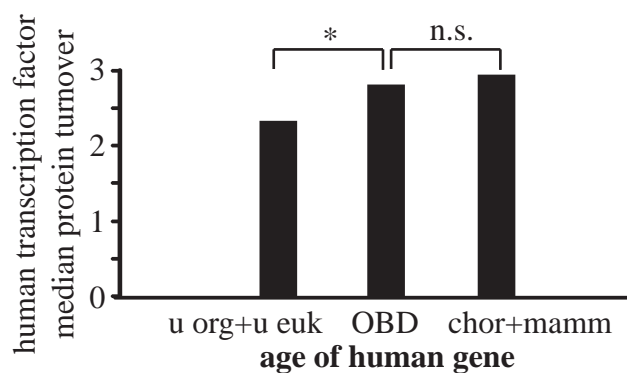

B

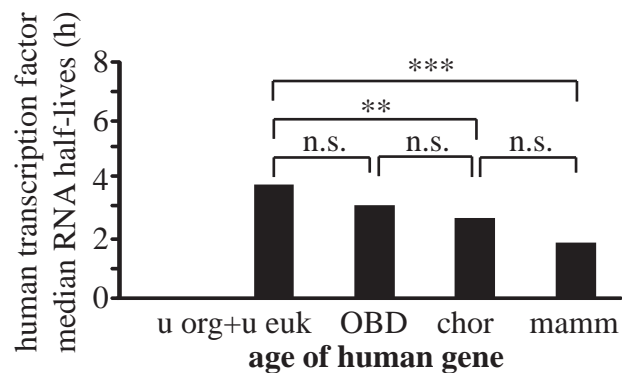

C

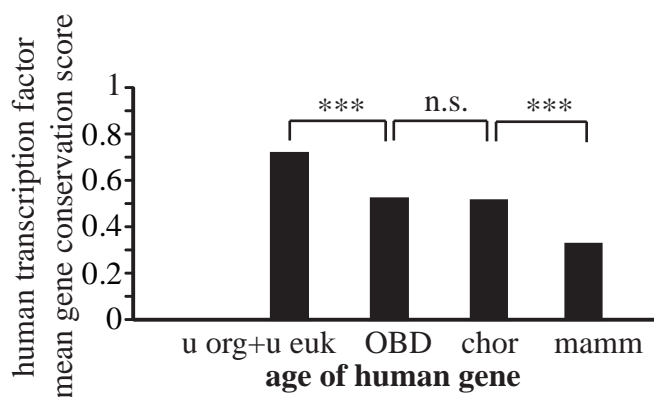

D

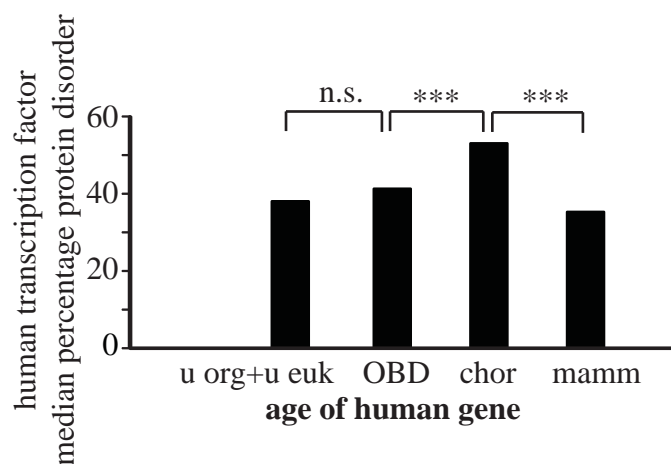

E

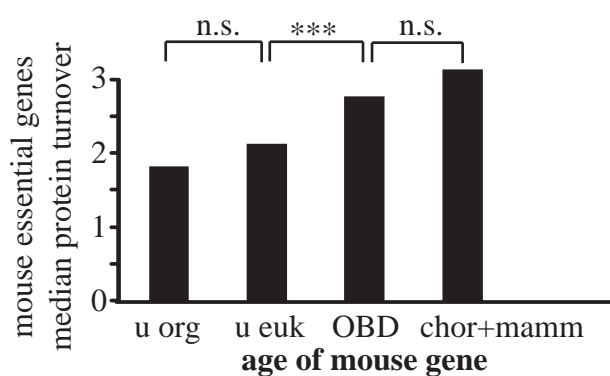

F

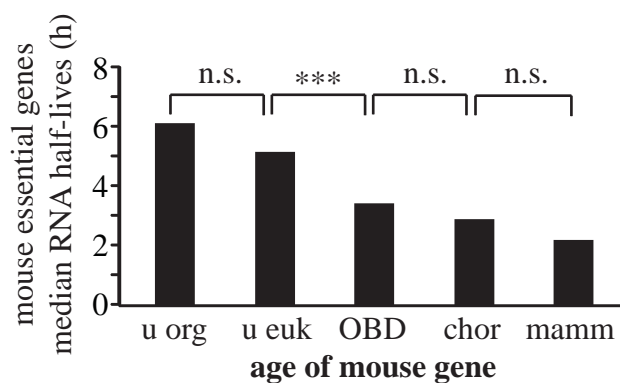

Supplementary Figure 4

Supplement: Supplementary file 4 — Additional file 4: Figure S4. Age-dependent changes of human transcription factors and mouse essential genes. (a) Median protein turnover for taxonomic groups of transcription factors. Number of proteins: u org+ u euk=34, OBD=34, chor+ mamm=23. Groups with less than 10 genes were added to the neighboring group (also in b,c,d) (b) Median mRNA half-lives for taxonomic groups of transcription factors. Number of different mRNA species: u org+ u euk=51, OBD=172, chor=175, mamm=89.(c) Mean gene conservation score for taxonomic groups of transcription factors. Number of genes: u org+u euk=79, OBD=471, chor=509, mamm=241. (d) Median level of protein disorder for taxonomic groups of transcription factors. Number of proteins: prok+ u euk=82, OBD=531, chor=512, mamm=322. (e) Median protein turnover for taxonomic groups of mouse essential genes. Number of proteins: u org=92, u euk=168, OBD=129, chor+ mamm=73. (f) Median mRNA half-lives for taxonomic groups of mouse essential genes. Number of different mRNA species: u org=133, u euk=296, OBD=327, chor=222, mamm=13. (ANOVA, Bonferroni post-hoc analysis for all figures). [file 12864_2019_6371_MOESM4_ESM.pdf]
