# Supplementary material for: Hypothesis: protein and RNA attributes are continuously optimized over time
Source: BMC Genomics. 2019 Dec 23;20:1012. doi: 10.1186/s12864-019-6371-0 (PMC6929361; doi:10.1186/s12864-019-6371-0)
Supplement: Supplementary file 5 — Additional file 5: Table S3 and Figure S5. Table S3: Median protein turnover values for GO categories based on mass spectrometry proteomics data from arrested human HeLa and differentiated mouse muscle C2C12 cells. Probability indicates difference to average turnover of all proteins. Figure S5: Evolutionary changes of protein turnover between human and mouse. (a) Graphical representation of Supplementary Table 3. (b) Median peptide turnover values of the ‘histone deacetylase and nucleosome remodeling activities complex’ proteins. (c) Grouping of proteins into equal size bins of 210 and quantification of homologous proteins with a turnover increase from human to mouse. [file 12864_2019_6371_MOESM5_ESM.pdf]

Complex	Median human	Median mouse	# Proteins
**20S core proteasome**	**1.72**	**1.46**	**14**
17S U2 snRNP/SF3b (spliceosome)	1.97	1.84	14
macromolecular tRNA synthetase complex	1.72	1.62	9
chaperonin-containing t-complex	1.78	1.73	9
Mammalian translation initiation factor 3 (eIF3)	2.14	1.84	10
**COP9 signalosome complex**	**1.76**	**1.56**	**8**
**19S proteasome regulatory cap**	**1.84**	**1.57**	**9**
**COPI coatomer complex**	**1.98**	**1.61**	**6**
**ER complex that binds nascent proteins**	**1.88**	**1.54**	**5**
G-protein complex	2.60	2.67	6
messenger ribonucleoprotein complex	1.85	1.77	14
**histone deacetylase and nucleosome remodeling activities complex**	**2.60**	**3.10**	**5**
DNA-dependent protein kinase complex	1.80	2.27	4
double-stranded RNA activated protein kinase PKR complex	1.64	1.77	5
Nonsense-Mediated mRNA Decay complex	2.01	2.26	5
Secretory glycoprotein associated ER chaperone complex	1.89	1.49	4

A

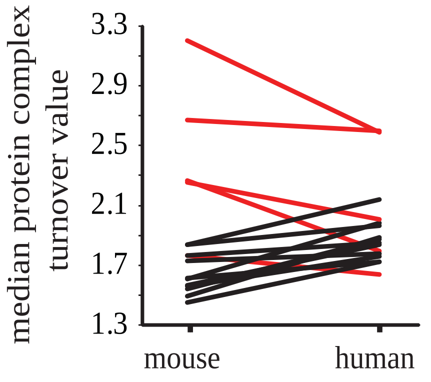

B

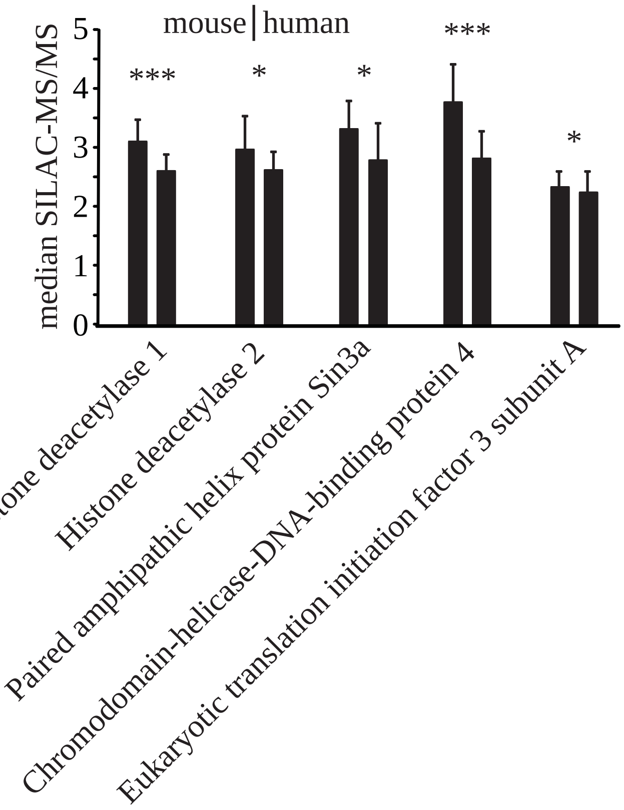

C

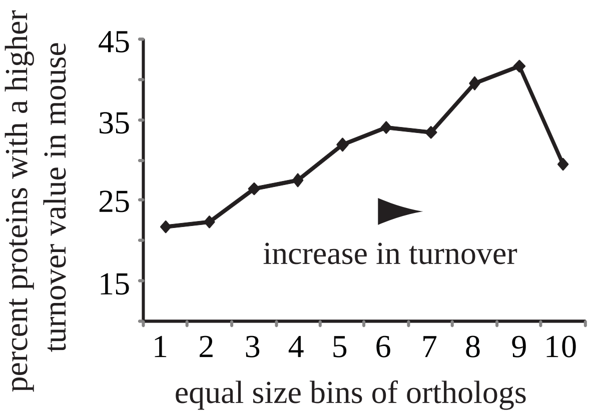

Supplementary Table 3
Supplementary Figure 5
